# Association of Maternal Social Relationships With Cognitive Development in Early Childhood

**DOI:** 10.1001/jamanetworkopen.2018.6963

**Published:** 2019-01-11

**Authors:** Eun Kyong Shin, Kaja LeWinn, Nicole Bush, Frances A. Tylavsky, Robert Lowell Davis, Arash Shaban-Nejad

**Affiliations:** 1 University of Tennessee Health Science Center–Oak Ridge National Laboratory Center for Biomedical Informatics, Department of Pediatrics, University of Tennessee Health Science Center, Memphis; 2 Child and Adolescent Division, Department of Psychiatry, Weill Institute for Neurosciences, University of California, San Francisco; 3 Division of Developmental Medicine, Department of Pediatrics, University of California, San Francisco; 4 Division of Biostatistics and Epidemiology, Department of Preventive Medicine, University of Tennessee Health Science Center, Memphis

## Abstract

**Importance:**

This study examines how different types of social network structures are associated with early cognitive development in children.

**Objectives:**

To assess how social relationships and structures are associated with early cognitive development and to elucidate whether variations in the mother’s social networks alter a child’s early cognitive development patterns.

**Design, Setting, and Participants:**

This cohort study used data from 1082 mother-child pairs in the University of Tennessee Health Science Center–Conditions Affecting Neurocognitive Development and Learning and Early Childhood project to examine the association between networks of different levels of complexity (triad, family, and neighborhood) and child cognitive performance after adjustment for the mother’s IQ, birth weight, and age, and the father’s educational level. The final model was adjusted for the household poverty level. Data were collected from December 2006 through January 2014 and analyzed from October through November 2018.

**Exposures:**

The child-mother relationship, child-mother-father triad, family setting, child’s dwelling network, mother’s social support network, and neighborhood networks.

**Main Outcomes and Measures:**

Measure of cognitive development of the child using Bayley Scales of Infant Development (BSID) at 2 years of age.

**Results:**

Of 1082 participants, 544 (50.3%) were males and 703 (65.1%) were African American; the mean (SD) age was 2.08 (0.12) years. Large family size had a negative association with early cognitive development, with a mean 2.21-point decrease in BSID coefficient score (95% CI, 0.40 to 4.02; *P* = .01). Mother’s social support network size was positively associated early cognitive development, with a mean 0.40-point increase in BSID coefficient score (95% CI, 0.001 to 0.80; *P* = .05). Knowing many neighbors was not statistically significantly associated with early cognitive development, with a mean 1.39-point increase in BSID coefficient score (95% CI, −0.04 to 2.83; *P* = .06).

**Conclusions and Relevance:**

The findings suggest that maternal social relationships are associated with cognitive development in children and that social relationships beyond the mother-child-father triad are significantly associated with children’s cognitive development. This study investigates the environmental influences on child health outcomes and, specifically, how early cognitive development is associated with social networks for the primary caregiver.

## Key Points

**Question:**

How are social relationships and structures, such as dyads, families, and neighborhoods, associated with early cognitive development in children?

**Findings:**

In this cohort study of 1082 mother-child pairs, the mother’s social networks were significantly positively associated with early childhood cognitive development. Being in a large family network was significantly associated with lower cognitive performance.

**Meaning:**

The findings suggest that maternal social relationships are associated with cognitive development in children and that social relationships beyond the mother-child-father triad are significantly associated with cognitive development.

## Teaser

This cohort study of mother-child pairs from Tennessee investigates whether variations in maternal social networks are associated with cognitive development in children.

## Introduction

Social networks, broadly defined as interconnectedness with other people, can influence behavioral and health outcomes.^1,2,3^ The importance of social and relational environments for cognitive development and emotional well-being is well known.^4,5,6,7,8^ Networks of social support can attenuate psychologic stress and provide support to people experiencing neurotic symptoms.^9,10,11,12^ The progress that children make when forming healthy relationships during the period from birth to 5 years can have long-lasting benefits throughout their entire lives.^13^ In early childhood development, relationships between children and caregivers are crucial and play an important role in socialization.^14,15,16,17^ The social environment and relationships early in life are critical for children’s emotional, intellectual, and social development into adulthood and can considerably influence the child’s life-long adaptation strategy.^6,13,18,19,20,21^ Social networks channel benefits and risks associated with social health determinants, such as health-related knowledge, attitudes, and capacity to cope with adversities associated with social disadvantage.^1,4,6,22,23,24^

The network microsystem has been regarded as a critical domain for child development.^6,15^ Children’s early experience of relationships can influence a wide range of developmental outcomes,^7,15,25^ and the child-mother relationship is important in shaping early childhood development.^17,21,26,27^ From the child’s perspective, these intimate bonds form the basis for solid attachments and provide prototypes for adulthood and the basis for social interaction.^15,28,29,30,31^ The child-mother relationship is not the only determinant but is nested within larger social contexts. The father plays a significant role in children’s development,^17,25,32,33,34,35^ as do the number of siblings and household size.^36,37^ Social support, along with other aspects of the social networks surrounding the child-mother bond, can influence the child-mother relationship.^38,39^ For mothers, social support is significantly associated with lower maternal stress, which is correlated with better child development.^40,41,42,43^

Little is known about how different types of relationships, especially the multiple social networks of the mother, are associated with children’s cognitive development. Only a few studies^8,43^ have characterized a range of maternal social networks and examined their association with early childhood cognitive development. In this study, we examined the associations of multiple types of social relationships and structures, including the child-mother-father triad, family setting, and larger neighborhood network conditions, with early cognitive development. Within a large group of white and African American families in Memphis and Shelby County, Tennessee, we examined how social relationships and networks were associated with children’s cognitive development.

## Methods

### Data Source

This study followed Strengthening the Reporting of Observational Studies in Epidemiology (STROBE) reporting guideline.^44^ We used data from the University of Tennessee Health Science Center’s Conditions Affecting Neurocognitive Development and Learning in Early Childhood (CANDLE) project.^45^ Recruitment for CANDLE started from December 2006 through July 2011. A total of 1503 mothers with a low-risk pregnancy were recruited at 16 to 28 weeks’ gestation from 5 participating health care settings in Shelby County, Tennessee. For 1082 mothers, a measure of cognitive development of the child at age 2 was available, representing the final sample for analysis. In this subset, the race/ethnicity of participants reflected the sociodemographic characteristics of Shelby County. This study was approved by the University of Tennessee Health Science Center institutional review board and participant written informed consent was obtained. Data were collected from December 2006 through January 2014 and analyzed from October through November 2018.

### Variable Definitions

Table 1 summarizes the key variables used in the analysis and their descriptive distribution. Because our outcome variable was cognitive development at 2 years of age, we used the Bayley Scales of Infant Development (BSID), which is designed to measure the developmental functioning of young children and to identify potential developmental delays.^46,47^ The BSID^46^ is composed of 5 scales: cognitive (score range, 55-145), language (47-153), motor (46-154), socio-emotional (55-145), and adaptive behavior (40-160).

**Table 1.** Characteristics of 1082 Study Participants in CANDLE

Variable	Value^a^	BSID Score, Mean (SD)
BSID categories	1082 (100)	97.74 (12.87)
Sex		
Male	544 (50.3)	96.25 (12.73)
Female	538 (49.7)	99.26 (12.85)
Race/ethnicity		
African American	703 (65.1)	105.09 (13.33)
White	377 (34.9)	93.81 (10.75)
Poverty level		
Below FPL	405 (40.9)	93.51 (10.73)
At or above FPL	586 (59.1)	101.27 (13.44)
Father’s educational level		
Some high school or graduated high school	661 (63.0)	95.31 (11.58)
Some college	76 (7.2)	99.28 (14.67)
College and graduate school	312 (29.7)	103.17 (13.50)
Father’s cohabitation		
Father not living at home	421 (42.0)	94.53 (11.38)
Father living at home	570 (58.0)	100.61 (13.47)
Family size		
<6 People	813 (75.1)	99.19 (13.23)
≥6 People	269 (24.9)	93.36 (10.63)
Mother’s social network size, mean (SD)	3.49 (1.82)	NA
Neighborhood embeddedness		
Not knowing many people in the neighborhood	408 (41.2)	95.47 (11.46)
Knowing many people in the neighborhood	582 (58.8)	99.92 (13.65)
Mother’s WASI IQ, mean (SD)	96.11 (16.25)	NA
Birth weight, mean (SD), g	3266.04 (537.10)	NA
Mother’s age at child’s birth, mean (SD), y	26.55 (5.50)	NA

Abbreviations: BSID, Bayley Scales of Infant Development; CANDLE, Conditions Affecting Neurocognitive Development and Learning in Early Childhood; FPL, federal poverty level; NA, not applicable; WASI, Wechsler Abbreviated Scale of Intelligence.

^a^ Data are presented as number (percentage) of participants unless otherwise indicated.

Because of the availability of extensive information about child-mother relationships and their contexts in the CANDLE study, we systematically examined multiple layers of networks and their association with cognitive development in early childhood.

The framework of the stepwise network structures is presented in the Figure. We studied 4 network conditions: father’s cohabitation (triad), large family network (family), mother’s social support network (caregiver’s social support network), and neighborhood. Family network size was estimated using data on household size, including all adults and other children living with the child involved in the CANDLE study. The mean (SD) of the household size variable was 4.37 (1.51) people, and we defined a family of 6 or more as a large family network. The primary caregiver’s social network was defined by the mother’s self-reported social support network. The mothers participating in CANDLE reported 3 to 4 people they could rely on for help, with a mean (SD) of 3.49 (1.82) people. We also included an indicator variable that asked mothers if they knew many people in their neighborhood.

**Figure.**
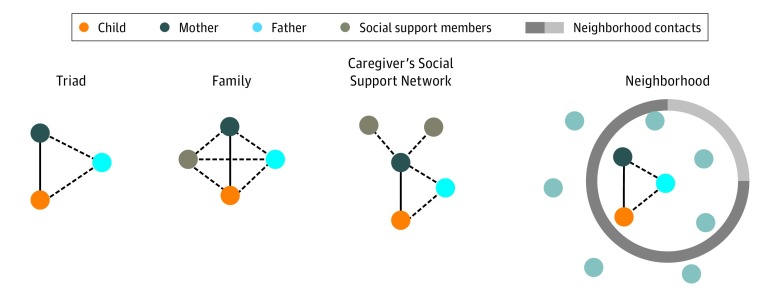
Stepwise Network Exposure Conditions The solid line between mother and child represents a tie that is always present, as in this study because children were recruited through their mother; dashed lines represent ties that may or may not exist.

### Statistical Analysis

We used multivariate robust regression models to study the associations of multiple social networks and cognitive development of 2-year-old children. To minimize the influence of outliers, we used robust regression methods.^48^ This approach allowed us to investigate how multiple layers of social network conditions are associated with cognitive development in early childhood. A 2-sided *P* ≤ .05 was set a priori to represent a statistically significant difference. We used Stata, version 14 (StataCorp) for statistical analysis.

We adjusted for several maternal and socioeconomic characteristics in a stepwise fashion. The first step included network variables.^32^ We included the following factors as independent variables in the model because of potential confounding: mother’s IQ, child’s birth weight, mother’s age, and father’s educational level. We originally tested other possible confounders, such as child sex, gestational age at birth, breastfeeding, birth weight, maternal smoking, and mother’s educational level. However, they were not included in the model because they did not substantively influence the coefficient of the main variables. The second model was adjusted for the same variables as the main model, but family poverty level was added.^49,50,51,52,53,54,55,56,57^

## Results

Of 1082 participants, 544 (50.3%) were males and 703 (65.1%) were African American; the mean (SD) age was 2.08 (0.12) years. The BSID score at 2 years of age ranged from 55 to 145 (mean [SD], 97.74 [12.87]). After adjustment for household poverty (Table 2), some social network characteristics were significantly associated with cognitive development. Mother’s social network was significantly associated with increased mean BSID coefficient scores (difference, 0.40; 95% CI, 0.001 to 0.80; *P* = .05), whereas living in a large family was associated with a 2.21-point decreased mean BSID coefficient score (95% CI, −4.02 to −0.40; *P* = .01). Father’s cohabitation (0.07; 95% CI, −1.58 to 1.73) and knowing many neighbors (1.39; 95% CI, −0.04 to 2.83; *P* = .06) were not significantly associated with an increased mean BSID coefficient score after controlling for poverty level.

**Table 2.** Stepwise Network Associations in Early Cognitive Development at 2 Years of Age

Characteristic	Model 1^a^		Model 2^b^	
	Coefficient Value (95% CI)^c^	*P* Value	Coefficient Value (95% CI)^c^	*P* Value
Father’s cohabitation	0.40 (−1.22 to 2.01)	NA	0.07 (−1.58 to 1.73)	NA
Large family	−2.41 (−4.21 to −0.61)	.01	−2.21 (−4.02 to −0.40)	.01
Mother’s social network	0.43 (0.03 to 0.83)	.04	0.40 (0.001 to 0.80)	.05
Neighborhood	1.44 (0.01 to 2.88)	.05	1.39 (−0.04 to 2.83)	.06
Below poverty level	NA	NA	−1.59 (−3.33 to 0.15)	NA

Abbreviation: NA, not applicable.

^a^ The following factors were induced as independent variables because of potential confounding: mother’s IQ, birth weight, mother’s age, and father’s educational level.

^b^ Same variables as model 1 plus household poverty level.

^c^ Coefficient values are defined as the difference in Bayley Scales of Infant Development scores.

## Discussion

The importance of the mother-child bond for child development has long been recognized.^17,21,26,27^ However, to our knowledge, the multiple layers of a mother’s social relationships beyond mother and child have not been simultaneously examined. Social relationships do not exist in isolation, and mother-child relationships are intertwined with other relationships, such as spouses, family or dwelling settings, and friendships. Our empirical analysis simultaneously investigated the association of multiple layers of the mothers’ social networks with children’s early cognitive development. Most social contacts and contexts of young children were determined by their primary caregiver’s social networks, who were often mothers in the CANDLE cohort.

In this study, we showed that a primary caregiver’s network conditions were significantly associated with early cognitive development in children. Network variables were significantly associated with early cognitive development after controlling for a number of biological and social confounders. Specifically, the mother’s social network seemed to have a beneficial association with the cognitive development of children, whereas family size had a negative association. Although father’s cohabitation has been suggested to be an important factor for early childhood cognitive development,^17,34,58^ after controlling for other social network conditions of the mother and other possible confounders, we did not find evidence for this result in our study. This might be because of the local context—Memphis is an economically disadvantaged area of the United States, which might mitigate an otherwise positive association of father’s cohabitation.

Many of our findings are consistent with previous studies^36,37,59^ of early cognitive development and add important evidence that social networks across several levels may be significantly associated with cognitive development in early childhood. Being raised in a large family (≥6 people) was significantly associated with lower cognitive performance, a finding that is also in line with previous studies of large families.^36,37^ However, past studies have also shown that family size can have advantages (ie, positive socialization) and disadvantages (ie, resource competition or limitation).^59^ Our findings about the negative association of family size may have been attributable to the limited attention and resources that a child received from the primary caregiver when faced with competing demands. Further investigation is needed to identify the mechanisms of this disadvantage.

We also reported results that large maternal social networks were positively associated with the cognitive development of children. Children of mothers who knew many people in the neighborhood had better cognitive development. It is possible that mothers who socialized locally provided children with more opportunities for playdates with other children or stimulation through more social activities. In addition, the primary caregiver’s social networks within the neighborhood may have buffered the association between reduced economic resources and child outcomes.^60^ Our results captured the importance of a community-based social life.

### Limitations

Our study has a number of limitations. We did not have information on relationship quality. Our measure of neighborhood embeddedness was based on subjective perception self-report. In addition, we only looked at relational embeddedness, not the physical or built neighborhood environment. Further research is needed to examine the nature of the association between neighborhood embeddedness, both physical and relational, and cognitive development in early childhood.

## Conclusions

The findings suggest that social relationships beyond the mother-child-father triad are significantly associated with children’s cognitive development and that maternal social relationships may be associated with the cognitive development of children.
